# The Influence of Heights of Power Arm for Controlled Anterior Teeth Movement in Sliding Mechanics: A 3D FEM Study

**DOI:** 10.7759/cureus.25976

**Published:** 2022-06-15

**Authors:** Soja Sara George, T.R. Jayaprakash Reddy, Sujan Kumar KV, Gagan Chaudhary, Umar Farooq, Vishnupriya Cherukuri, Chadawala Likitha

**Affiliations:** 1 Department of Orthodontics, MNR Dental College and Hospital, Telangana, IND; 2 Department of Dentistry, Dr. VRK Women's Medical College, Teaching Hospital and Research Center, Telangana, IND

**Keywords:** mini-screw, 3d finite element method, anterior teeth retraction, power arm, sliding mechanics

## Abstract

Introduction: The effectiveness of orthodontic treatment depends on precise control of the anterior teeth during retraction in sliding mechanics. That is why researchers were interested in finding the appropriate loading conditions for sliding mechanics to govern anterior teeth's movement, such as the height of retraction force application and its location on the arch wire.

Methods: A FEM study was conducted to evaluate the type of movement of anterior teeth during en-masse retraction by applying 200 gms of force to each side of the maxillary arch with power arms set at various levels (4 mm, 6 mm, 8 mm) mesial to canine from mini-screw placed at a height of 8 mm between maxillary first molar and second premolar. A 3D model (FEM) of the maxillary arch was constructed to study the amount of labio-lingual tipping and bodily displacement of maxillary central incisor achieved.

Results: When a 200-gm power was applied to the power arm, controlled lingual crown tipping was seen at the levels of 4 mm and 6 mm, in contrast to the level of 8 mm, where bodily displacement occurred, according to this evaluation.

Conclusion: In sliding mechanics, the height of the power arm plays an important role in obtaining controlled lingual crown tipping or bodily displacement during retraction of anterior teeth.

## Introduction

Anterior tooth retraction with sliding mechanics has been frequently employed for space closure after premolar extraction. The effectiveness of orthodontic treatment depends on the ability to precisely control the movement of the anterior teeth during space closure using sliding mechanics. Power arms coupled to the arch wire make this possible [1].

The power arm used to deliver retraction force at the anterior teeth's center of resistance (CR) has been lengthened to aid in movement. Sliding mechanics anterior teeth retraction may be performed with a wide range of vertical retraction pressures by attaching varying lengths of power arms to an arch wire [2,3]. Consequently, it is important to know and use the optimal power arm height for effective anterior tooth retraction. The introduction of mini-implants to orthodontic practice as an absolute anchorage device in clinical conditions where maximum anchorage is required for en masse retraction also made it possible to obtain an effective and controlled anterior tooth retraction with no anchor loss [4]. 

When it comes to the biomechanical aspects of orthodontic tooth movement [5], much research has employed laser holography, strain gauge methods, photoelasticity methods, and the finite element method (FEM). When it comes to studying the biomechanical and biological aspects of orthodontic tooth movement, finite element analysis is an excellent tool. Stress distributions and 3-D displacements may be calculated using the finite element method (FEM) in a wide range of systems with non-homogeneous physical features [6,7].

We wanted to see what kinds of tooth movements we might elicit by delivering 200gm of force to the power arm at various heights. The degree of labio-lingual tilting of the maxillary central incisor was determined using a 3-D finite element simulation of en-masse retraction in sliding mechanics utilizing a 19x 25 SS arch wire in a 0.022 MBT slot prescription.

## Materials and methods

Experimental conditions 

**Figure 1:**
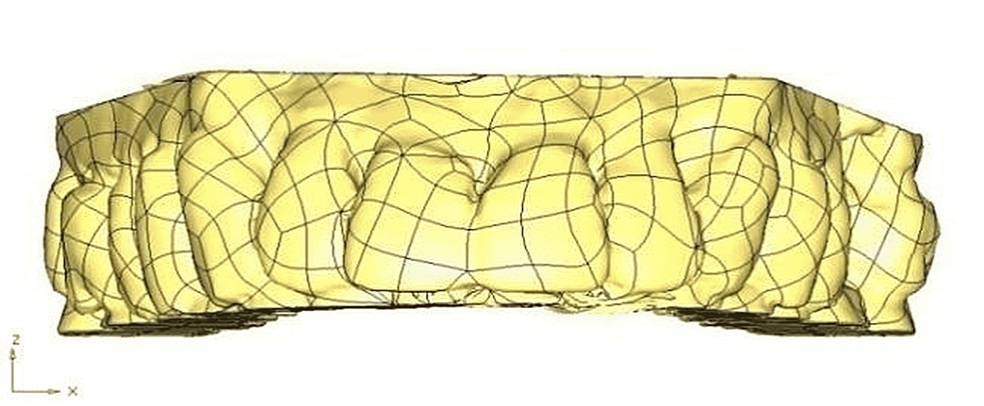
3D mesh of the area examined

**Figure 2:**
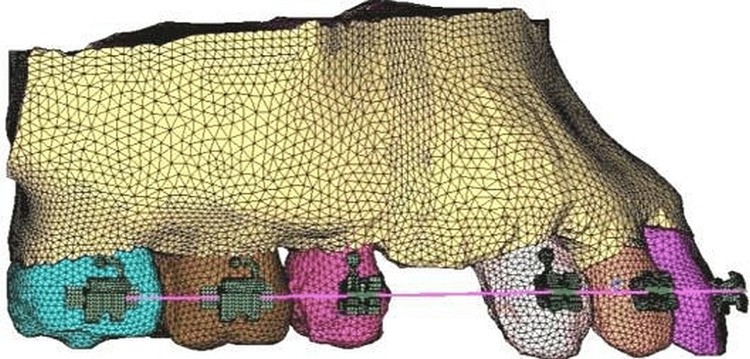
FEM model of the area constructed

A CT image of the maxilla from a skull was used to generate a model of the 12 maxillary front teeth and their supporting designs (PDL and alveolar bone). Digital data from the CT scans were saved as DICOM (electronic imaging and correspondence in drug) files and sent to a third party for analysis, where a numerical model was developed to treat the patient's disease. The numerical model was entirely converted to FEM using HyperMesh programming (Figures 1, 2).

**Figure 3:**
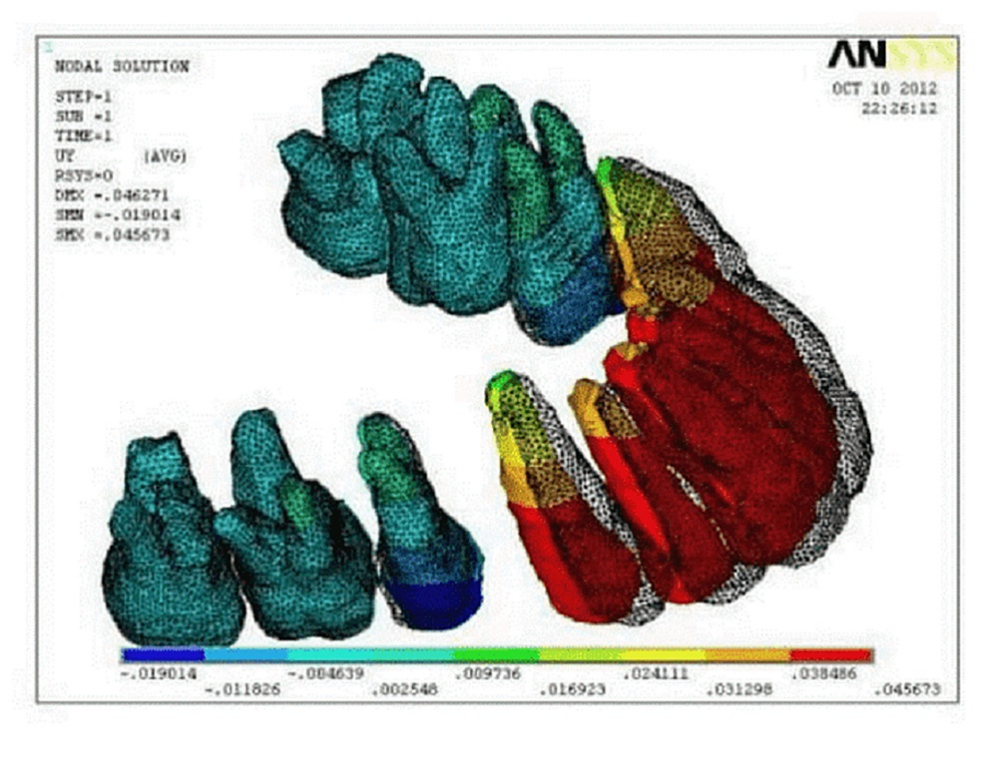
Ansys software image

The maxillary premolars are removed. Between the maxillary second premolar and the primary molar, titanium mini-implants of 1.2 mm in width and a length of 8 mm were placed buccally at an 8 mm height from the gingival margin (Figure 3). Ansys, a biomechanical evaluation tool, was used to simulate the maxillary 12 teeth's restricted portion to display.

Two power arms were attached mesial to the canine bilaterally and perpendicular to the arch wire. An appliance with 0.022 bracket slots and a 0.019 x 0.025-in stainless steel arch wire was generated. A retraction force of 200 gm was applied bilaterally from the mini-implant model to three heights of retraction hook placed mesial to canine (4, 6, and 8mm).

Based on these 3D solid models, a finite-element mesh was created to make a node-to-node connection between tooth, alveolar bone, and PDL. The amounts of tooth movements were calculated at each nodal point using HyperMesh (Paterna, Valencia) and Ansys software (Pennsylvania, USA) for generating models.

Material parameters

To accurately model the behavior of an object, a FEM must have the right material characteristics assigned to it. Teeth, periodontal ligament, alveolar bone, and stainless steel were all deemed homogeneous and isotropic in this investigation. The Young's Modulus (also known as the modulus of elasticity) and the Poisson's Ratio 8 were the material qualities given (Table 1).

**Table 1:** Young’s modulus and Poisson’s ratio for various materials used in this study.

Material	Young’s modulus	Poisson’s ratio
Tooth	2.07x10^3^ kgf/mm^2^	0.30
Compact bone	1.37x10^3^ kgf/mm^2^	0.30
Cancellous bone	8.00x10^2^ kgf/mm^2^	0.30
Archwire/powerarm/bracket/ Ti alloy	107.0	0.30

## Results

In the present FEM study, the results demonstrated the amount of labio-lingual tipping achieved in the anterior teeth with different power arm lengths. In order to express the link between the kind of movement and the length of the retraction hook, each tooth's rotational angle was recorded. Based on the determined rotation (tipping) angle, a graph was created and viewed. A graph was plotted with a height of retraction (in millimeters) on X-axis and rotation angle (in degrees) on the Y-axis. A positive sign indicates a lingual crown tipping and a negative sign indicates lingual root tipping. 

A controlled tilting of the anterior teeth happened when a force of 200 gm was given to a 4mm height power arm, which was positioned mesial to the canine. The rotation angle was -0.1 degrees when the retraction hook's height was increased to 8mm. That signifies the rotation has gone from the lingual crown tipping (positive indication) to the lingual root tipping (negative sign).

Using a retraction force of 200 gm, millimeters of crown and root apex displacement were measured at each height of the retraction hook in the sagittal plane for the front teeth. According to the results, the retraction hook's height was shown to be responsible for a displacement of the crown and root of 0.033 millimeters, whereas, at a distance of 6 millimeters, the displacement was 0.034 millimeters. When the retraction hook reached 8 mm high, the crown and root were both displaced by the same amount of 0.034mm (Table 2).

**Table 2:** Displacements in millimeters for the anterior teeth when retraction force of 200 gm was applied.

Tooth Displacement	Anterior Retraction Hook Heights			
	TAD=8mm from CEJ	4mm	6mm	8mm
Central Incisor	Crown	0.033mm	0.034mm	0.034mm
	Root	0.023mm	0.024mm	0.035mm
Lateral Incisor	Crown	0.034mm	0.034mm	0.034mm
	Root	0.022mm	0.03mm	0.034mm
Canine	Crown	0.033mm	0.034mm	0.034mm
	Root	0.011mm	0.017mm	0.018mm

So, when a retraction force of 200 grams was given to the four and six-millimeter hooks, root displacement happened more often than a crown dislocation. There was a bodily movement of anterior teeth, i.e., the same amount of crown and root displacement seen for retraction hook height of 8mm.

## Discussion

Sliding mechanics is the commonly used mechanics for space closing after premolar extraction. Orthodontic mini-implants are used as a skeletal anchorage, which provides absolute stability to the segments by providing resistance toward undesirable reactionary tooth movements [9]. An 8-mm distance from the gingival margin was chosen for the placement of mini-implants in this study, which was shown to be appropriate for passing of force vector to the center of resistance. In the FEM model, Ashekar et al. [4] showed that a low mini-implant placement (6 mm) leads the anterior teeth to the tip, and a mid- mini-implant placement (8 mm) results in bodily movement, and a high mini-implant placement (10 mm) produces an intrusion. But Chetan et al. suggested that the mini-implants position in the vertical plane will influence the type of tooth movement. Ghannam et al. [8] observed fewer tipping moments when the implant was placed 8mm from arch wire than when it was placed at 4.5mm height. In the present study, the mini-implant was placed 8mm from the gingival margin because it made the force vector pass as close to the center resistance which brings a bodily retraction, and also placing coronal to this height might result in perforation of PDL, sinus or root damage [9].

By Story and Smith [10], an optimal power of 175 to 300 gm (1.75 to 3.0 N) was estimated for considerable canine withdrawal, and an appropriate power framework is important for an adequate organic response in the periodontal tendon. Lee predicted that a pressure range of 150-260 gm/cm2 would be necessary to achieve optimal tooth growth. Ruenpol et al. [9] used 200gm of power for withdrawal in their study and obtained development similar to the outcome of our present research. As shown in a study by Ren et al., it is impossible to identify an edge for force extent that would influence orthodontic tooth movement, as demonstrated in a study published in the Journal of Dental Research [11].

Lingual crown or root tipping or bodily movement are the forms of tooth movement that are influenced by center of resistance and the line of force application [12]. The height of the retraction hook might change the center of rotation of the anterior teeth during space closure in sliding mechanics. By attaching different lengths of retraction hook onto the arch wire, different types of tooth movement may be achieved [12,13].

During the present evaluation, the direction changed from lingual crown tipping to lingual root tipping because of the increase in the amount of the withdrawal catch. It was shown that lingual crown tipping (rotation angle is positive) was obtained for 4mm and 6mm retraction hooks, and lingual root tipping (negative rotation angle) was obtained for 8 mm retraction hook. It was found that when 200gm of force was applied to a retraction hook of 8mm placed mesial to canine, crown and root displacement occurred at the same rate, which indicated bodily retraction of maxillary teeth.

Limitations

Due to the complexity of force systems used in an orthodontic patient, the finite element (FE) method allows the analytical application of different force systems at any point and direction. The finite element method could be a very versatile method of studying the physical behavior of elements; however, biology is hard to predict and should be taken into consideration. An idealized geometric shape was used for the periodontal ligament and tooth roots; physical attributes were considered to be homogeneous, isotropic, and linear in the ideal model. This approach may still be utilized to characterize stress in the periodontal ligament (PDL) and adjacent alveolar bone, despite these limitations.

Further studies using FEM, including variables affecting biomechanical behavior of tooth movement and anatomic parameters, are still needed.

## Conclusions

It can be concluded that bodily retraction occurred when a force of 200gm was applied to a retraction hook of 8mm height, and controlled tipping occurred when lesser height retraction hooks (4mm, 6mm) are used. Uncontrolled tipping reduced as the power arm's height increased, and body movement was possible while retraction of anterior teeth utilizing mini-implant anchoring was performed. To obtain better control of the inclination of anterior teeth in sliding mechanics, orthodontists put a retraction hook height of 8 mm on an arch wire placed between the lateral canine and the incisor.
